# Forced IDO1 expression in dendritic cells restores immunoregulatory signalling in autoimmune diabetes

**DOI:** 10.1111/jcmm.12360

**Published:** 2014-09-12

**Authors:** Maria Teresa Pallotta, Ciriana Orabona, Roberta Bianchi, Carmine Vacca, Francesca Fallarino, Maria Laura Belladonna, Claudia Volpi, Giada Mondanelli, Marco Gargaro, Massimo Allegrucci, Vincenzo Nicola Talesa, Paolo Puccetti, Ursula Grohmann

**Affiliations:** Department of Experimental Medicine, University of PerugiaPerugia, Italy

**Keywords:** IDO1, tryptophan catabolism, autoimmune diabetes, plasmacytoid dendritic cells, immune regulation, non-canonical NF-κB, non-obese diabetic (NOD) mice

## Abstract

Indoleamine 2,3-dioxygenase (IDO1), a tryptophan catabolizing enzyme, is recognized as an authentic regulator of immunity in several physiopathologic conditions. We have recently demonstrated that IDO1 does not merely degrade tryptophan and produce immunoregulatory kynurenines, but it also acts as a signal-transducing molecule, independently of its enzymic function. IDO1 signalling activity is triggered in plasmacytoid dendritic cells (pDCs) by transforming growth factor-β (TGF-β), an event that requires the non-canonical NF-κB pathway and induces long-lasting IDO1 expression and autocrine TGF-β production in a positive feedback loop, thus sustaining a stably regulatory phenotype in pDCs. IDO1 expression and catalytic function are defective in pDCs from non-obese diabetic (NOD) mice, a prototypic model of autoimmune diabetes. In the present study, we found that TGF-β failed to activate IDO1 signalling function as well as up-regulate IDO1 expression in NOD pDCs. Moreover, TGF-β-treated pDCs failed to exert immunosuppressive properties *in vivo*. Nevertheless, transfection of NOD pDCs with *Ido1* prior to TGF-β treatment resulted in activation of the *Ido1* promoter and induction of non-canonical NF-κB and TGF-β, as well as decreased production of the pro-inflammatory cytokines, interleukin 6 (IL-6) and tumour necrosis factor-α (TNF-α). Overexpression of IDO1 in TGF-β-treated NOD pDCs also resulted in pDC ability to suppress the *in vivo* presentation of a pancreatic β-cell auto-antigen. Thus, our data suggest that a correction of IDO1 expression may restore its dual function and thus represent a proper therapeutic manoeuvre in this autoimmune setting.

## Introduction

Type 1 diabetes (T1D) is an autoimmune disorder whereby autoreactive T cells destroy insulin-producing cells in pancreatic islets. The genetically diabetes-prone non-obese (NOD) mouse strain is a prototypic model of the human disease. The predisposition of NOD mice to develop autoimmunity is presumably the result of defects in both peripheral and central tolerance mechanisms 1. Several abnormalities have been described in NOD mice, including impaired expression of cytotoxic T lymphocyte antigen 4 (CTLA-4) 2, interleukin (IL)-12 production 3, peroxynitrite formation 4 and aberrant accessory cell function 5. Various treatments that mobilize tolerogenic forces and/or exposure to specific environmental stimuli have been shown to protect NOD mice from diabetes onset 6–9.

Plasmacytoid DCs (pDCs) represent a rare yet extremely important subset of DCs specialized in the secretion of type I interferon (IFN-α and IFN-β) in response to viruses 10. They are involved both in protective immunity and in tolerance induction and participate in adaptive immune responses by directing the differentiation of T cells and/or by suppressing T-cell responses, depending on the stage of pDC maturation and environmental needs 11–15. In T1D, owing to their role as regulators of T cell immunity, pDCs may impact the functional balance between pathogenic T cells and regulatory T cells (Tregs). Overall, on the one hand, pDCs likely present β-cell–auto-antigen–autoantibody complexes to the relevant autoreactive T cells 16. On the other, pDCs could also be protective, mainly *via* expression of various molecules fostering tolerance induction, among which is indoleamine 2,3-dioxygenase 1 (IDO1) 17,18.

IDO1 is regarded as a most versatile component of immunoregulatory loops, in both innate and acquired immunity. It catalyzes the first and rate-limiting step of tryptophan catabolism along a specific pathway, resulting in a series of immunoactive metabolites collectively known as kynurenines. NOD mice are defective in IDO1 expression, and NOD pDCs fail to up-regulate IDO1 in response to inflammatory stimuli such as IFN-γ, one of the most potent inducers of IDO1 expression and catalytic function 6,9,19.

In pDCs exposed to TGF-β, IDO1 becomes, instead, phosphorylated and it mostly acts as a signalling molecule, participating in a feedforward loop that enhances its own expression and that of TGF-β, thus amplifying and spreading tolerance 20,21. The TGF-β–dependent signalling function of IDO1 has not been examined in NOD mice as yet. In the present study, we investigated IDO1 responsiveness to TGF-β in NOD mice. Much like IFN-γ in IDO1 expression, TGF-β was unable to induce IDO1 regulatory function in this setting. However, its overexpression in NOD pDCs rescued much of the cells' tolerogenic potential both *in vitro* and *in vivo*.

## Materials and methods

### Mice

Female C57BL/6, BALB/c and NOD/MrkTac female mice, aged 8–12 weeks, were purchased from Charles River Breeding Laboratories (Calco, Milan, Italy) and Taconic (Albany, NY, USA), respectively. All animals were housed and fed under specific pathogen-free conditions. All *in vivo* studies were in compliance with national (Italian Approved Animal Welfare Assurance A-3143-01) and Perugia University Animal Care and Use Committee guidelines.

### DC purification, treatments and transfections

All purification procedures for pDCs and CD8^−^ DCs (hereafter referred to as cDCs, for conventional DCs) have been described 19,20,22–25. Purified pDCs were exposed for 24 hrs at 37°C to recombinant TGF-β (R&D Systems, Minneapolis, MN, USA) at the concentration of 20 ng/ml in the presence or absence of l/d 1-methyl-tryptophan (1-MT; Sigma-Aldrich, St. Louis, MO, USA), a standard IDO1 inhibitor, at the concentration of 4 μM.

For silencing *Ido1*, gene-specific small interfering RNA (siRNA) was predesigned on the basis of the gene sequence and was synthesized by Ambion Life Technologies (Carlsbad, CA, USA), which also supplied the negative control siRNA. Transfection of pDCs was as described 20,25. Plasmid constructs coding for wild-type IDO1 (wtIDO1) or IDO1 mutants (*i.e*., IDO.Y_115_FY_253_F, lacking both phosphorylable tyrosines present in ITIM1 and ITIM2 and IDO.H_350_A, lacking the histidine required for catalytic activity) were generated as described 20. Because immunostimulatory sequences present in plasmid DNA (*i.e*., unmethylated CpG motifs) may produce non-specific effects, particularly in cells such as pDCs (*i.e*., expressing high levels of toll-like receptor 9), pDCs were transfected by means of mRNAs, as described 24. Briefly, plasmids were linearized, purified by using a Geneprep kit (Ambion Life Technologies) and used as templates for the *in vitro* transcription reaction by using the mMESSAGE mMACHINE T7 Ultra Kit (Ambion). Concentration and quality of *in vitro*-transcribed mRNAs were assessed by spectrophotometry and agarose gel electrophoresis. For transfection, mRNA (2 μg) in 30 μl of transfection buffer (20 mM HEPES, 150 mM NaCl, pH 7.4) were pipetted into a sterile Eppendorf tube. In a separate polystyrene tube, 6.7 μl of 1,2 dioleoyl-3-trimethylammonium-propane was mixed with 30 μl of transfection buffer, and then both solutions were mixed gently by pipetting several times. After incubation at room temperature for 20 min., the mixture was added to 1 ml of complete medium containing 10^6^ pDCs and incubated for 24 hrs at 37°C in the presence of TGF-β or medium alone. Cells were then recovered, washed and immediately used for *in vitro* and *in vivo* experiments. Control treatments consisted of cells subjected to control mRNA obtained from the pTRI-Xef plasmid (supplied by the manufacturer) containing the *Xenopus* elongation factor 1 gene, which codes for a 50.2 kD protein 24.

### Real-time PCR

Real-time PCR analysis was done as described 20,22,26 with primers specific for *Ido1* and *Tgfb1*. Results are presented as the ratio of gene expression to *Gapdh* expression, as determined by the relative quantification method (change in cycle threshold).

### Western blot analysis

IDO1 expression was investigated in pDCs by immunoblot with a rabbit monoclonal anti-mouse IDO antibody (cv152) raised in our laboratory 27. Anti–β-tubulin antibody (Sigma-Aldrich) was used as a normalizer. Analysis of p100 and p52 expression was performed in whole cell lysates of pDCs by using anti-p100/p52 antibody (Cell Signaling Technology, Danvers, MA, USA) 25.

### ELISA and cytofluorometric analyses

Mouse IL-6, IL-10, IL-27, TNF-α and IFN-α were measured in culture supernatants by ELISA with specific kits (eBioscience, Inc., San Diego, CA, USA; Promega Italia, S.r.l., Milano, Italy; and pbl Assay Science, Piscataway, NJ, USA). An ELISA-based TransAM Flexi NF-κB Family Kit (Active Motif, Rixensart, Belgium) was used to monitor activity of NF-κB family members, as described 9,20,25. Cytofluorometric assays were conducted by using FITC-labelled anti-latency-associated peptide (LAP) TGF-β1 (clone TWA-2F8; BioLegend, San Diego, CA, USA) and PE-labelled anti-B220 antibodies (BD Pharmingen, San Diego, CA, USA) as described 28.

### Luciferase and kynurenine assays

Activation of the *Ido1* promoter was evaluated in pDCs transfected with a firefly luciferase construct of the *Ido1* promoter, as described 20. Briefly, the plasmid mIDOprom900-luc (30 μg) 29, which contains the mouse *Ido1* promoter (900 bp) and 70 nucleotides of non-coding sequence in *Ido1* exon 1 upstream of the firefly luciferase coding sequence, was transferred by electroporation into pDCs in Opti-MEM containing Glutamax (Gibco Life Technologies, Carlsbad, CA, USA). The renilla reporter plasmid pRL-TK (1 μg; Promega) was transferred by electroporation as an internal control of the transfection process. After incubation for 1 hr at 37°C, cells were transfected with the *Ido1-*encoding or irrelevant mRNA. Luciferase activity was assayed with the Dual Luciferase Reporter Assay Kit (Promega). The functional activity of IDO1 was measured *in vitro* in terms of the ability to metabolize tryptophan to l-kynurenine, whose concentration was measured by high-performance liquid chromatography in culture supernatants at 16 hrs after the addition of 100 μM tryptophan for the final 8 hrs 22,27.

### Skin test assay

A skin test assay was used for measuring major histocompatibility complex class I–restricted delayed-type hypersensitivity in response to challenge in the footpad with the IGRP or HY synthetic peptide, as described 6,19,20,28, using 12 week-old NOD, BALB/c or C57BL/6 mice as recipients, respectively. The H-2K^d^-restricted IGRP peptide (KYNKANAFL) is a diabetogenic autoantigen in NOD mice but is also recognized by H-2^d^-expressing BALB/c animals 20. The H-2D^b^-restricted HY peptide (WMHHNMDLI) contains the immunodominant epitope of the male mouse-specific minor transplantation antigen and is therefore recognized by CD8^+^ T cells in C57BL/6 female mice 20,25. The response to challenge in the footpad with the eliciting peptide was measured at 2 weeks, and results are presented as the weight of peptide-injected footpad relative to that of the vehicle-injected counterpart 6,19,20,28.

### Statistical analysis

Student's *t*-test was used for analysis of the results of *in vitro* studies. In the *in vivo* skin test assay, Student's *t*-test was used for statistical analysis by comparison of the mean weight of experimental footpads with that of their control, saline-injected counterparts 30,31. At least six mice per group per experiment were used, as computed by power analysis to yield a power of at least 80% with an α-value of 0.05 20,25.

## Results

### TGF-β fails to induce IDO1-dependent, immunosuppressive properties in pDCs from pre-diabetic NOD mice

Murine DCs present antigens in an immunogenic or tolerogenic fashion, the distinction depending either on the occurrence of specialized DC subsets or on the maturation or activation state of the DC. Although DC subsets may be programmed to direct either tolerance or immunity, appropriate environmental stimulation will result in complete flexibility of a basic programme 26,28. Relevant in this regard, we have previously obtained evidence that the skin test assay represents a reliable means of discriminating between the immunogenic and immunosuppressive potential of distinct DC subsets conditioned with different stimuli 6,19,20,22,24–26,28,30. In particular, while untreated cDCs from the spleens of conventional mice (*i.e*., C57BL/6 and BALB/c) present poorly immunogenic peptide antigens in a stimulatory fashion, pDCs, which are weakly immunogenic under basal conditions, acquire potent immunosuppressive effects when treated with TGF-β, such that they will prevent host priming by cotransferred immunogenic cDCs 20.

**Figure 1:**
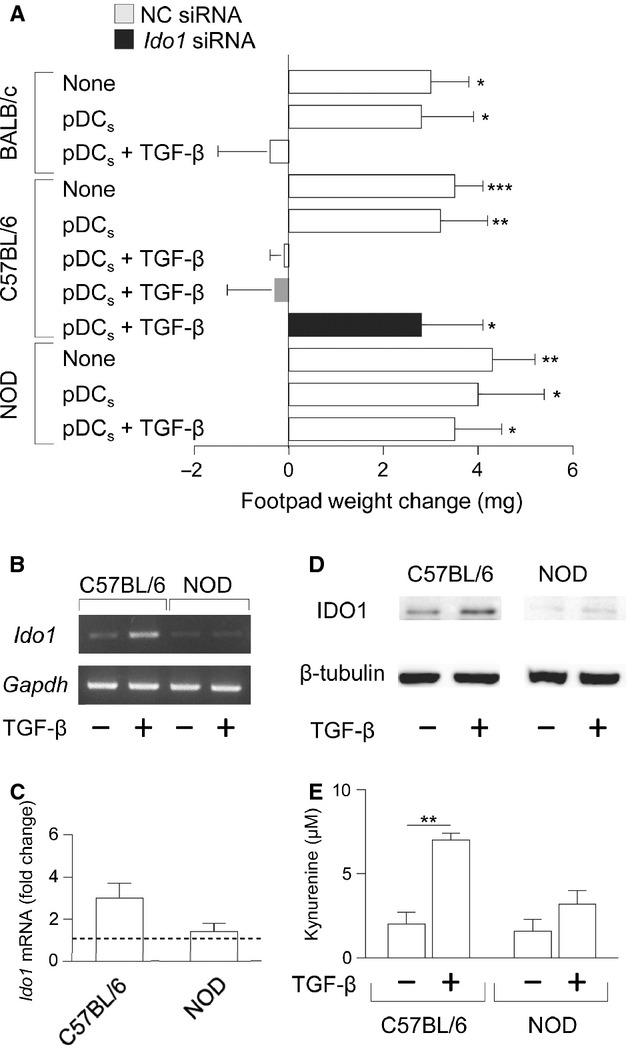
Transforming growth factor-β (TGF-β) fails to confer suppressive properties and induce IDO1 expression in pDCs from NOD mice. *In vivo* suppressive activity of TGF-β–conditioned pDCs from C57BL/6, BALB/c and NOD mice (**A**). Purified splenic cDCs and pDCs were pulsed for 2 hr with the HY (C57BL/6 groups) or IGRP (BALB/c and NOD) peptide and transferred into syngeneic recipient mice to be assayed for skin reactivity to the eliciting peptide. The cDC fraction was used in combination with 5% pDCs, pre-conditioned by TGF-β or medium alone, that were left untransfected, transfected with siRNA containing a scrambled sequence (negative control, NC) or targeting *Ido1* (only in C57BL/6 cells). Analysis of skin reactivity of recipient mice to the eliciting peptide at 15 d is presented as change in footpad weight. C57BL/6 and NOD pDCs were incubated with medium alone (control) or TGF-β for 16 hrs and *Ido1* mRNA was either qualitatively (**B**) or quantitatively (**C**) analyzed by conventional or real-time PCR, respectively, by using *Gapdh* normalization. In (**C**), data are presented as normalized transcript expression in the samples relative to normalized transcript expression in the control culture (that is, fold change = 1, dotted line). (**D**) C57BL/6 and NOD pDCs were treated with TGF-β or medium alone for 24 hrs and IDO1 expression was assessed by immunoblot analysis, by using an IDO1-specific antibody. (**E**) IDO1 catalytic activity in C57BL/6 and NOD pDCs left untreated or treated for 24 hrs with TGF-β was assessed as l-kynurenine in culture supernatants. *P < 0.05*,* **P < 0.01 and ***P < 0.001 (Student's *t*-test). Results are representative of three (**A**,**B** and **C**), five (**D**) and four (**E**) experiments (mean ± SD in **A**,**B** and **D**).

We therefore performed a skin test assay to evaluate whether TGF-β could induce an IDO1-dependent, immunoregulatory phenotype in splenic pDCs from NOD mice. Mice were sensitized with cDCs, administered alone or in combination with a minority fraction of pDCs (5% of the final cell mixture), left untreated or pre-treated for 24 hrs with TGF-β. Both cDCs and pDCs were pulsed with either the IGRP (for NOD and BALB/c DC donors and recipients) or the HY peptide (C57BL/6). After 2 weeks, reactivity was assessed by intrafootpad challenge with the solubilized peptide (the same as in priming), in the absence of DCs. As expected 20, the priming ability of cDCs was not affected by the presence of untreated pDCs in both conventional C57BL/6 and BALB/c mice (Fig.1A). Yet, cotransfer of cDCs with pDCs treated with TGF-β caused suppression of both HY-specific (C57BL/6) and IGRP-specific (BALB/c) reactivity, an effect abrogated by siRNA targeting *Ido1* but not by control siRNA (Fig.1A). In contrast, in NOD mice, the priming ability of cDCs was unaffected by TGF-β-pre-treated pDCs, with no difference relative to untreated pDCs (Fig.1A).

We next evaluated whether the lack of immunosuppressive effects by TGF-β in NOD pDCs could be ascribed to inability of the cytokine to correct the defective IDO1 expression in this autoimmunity-prone strain. We compared IDO1 transcripts and protein as well as production of l-kynurenine, the main IDO1 product, in pDCs from NOD *versus* C57BL/6 mice. In C57BL/6 pDCs, TGF-β up-regulated both IDO1 transcripts (Fig.1B and C) and protein expression (Fig.1D), and it induced a significant release of l-kynurenine in culture supernatants (Fig.1E). In NOD pDCs, the cytokine hardly increased IDO1 expression in terms of both transcripts and protein, which were almost negligible under basal conditions (Fig.1B–D), as observed previously 9. Moreover, l-kynurenine levels did not significantly increase in NOD TGF-β-treated pDCs as compared to untreated cells (Fig.1E).

Thus our data, besides indicating a global defect in IDO1 expression and function in NOD mice 6,9,19, further suggest that TGF-β fails to trigger IDO1 expression as well as IDO1-dependent signalling events, that would result in durable IDO1 activity in the pDCs.

### Forced IDO1 expression restores immunosuppressive effects of TGF-β in NOD pDCs

Triggering of IDO1 signalling in C57BL/6 pDCs by TGF-β strictly requires a very early event – IDO1 ITIM phosphorylation – by Fyn, a Src kinase highly expressed in pDCs from conventional strains of mice 20. This observation, therefore, implies that a basal level of IDO1 protein is necessary to allow the cytokine to trigger the phosphorylating events and, consequently, IDO1 signalling in pDCs. Because Fyn is also highly expressed in pDCs from NOD mice at all stages of the diabetes disease (data not shown), we reasoned that the failure of TGF-β to trigger IDO1-dependent signalling events in NOD pDCs could be ascribed to the hardly detectable amounts of IDO1 protein under basal conditions (Fig.1D and Ref. 9).

**Figure 2:**
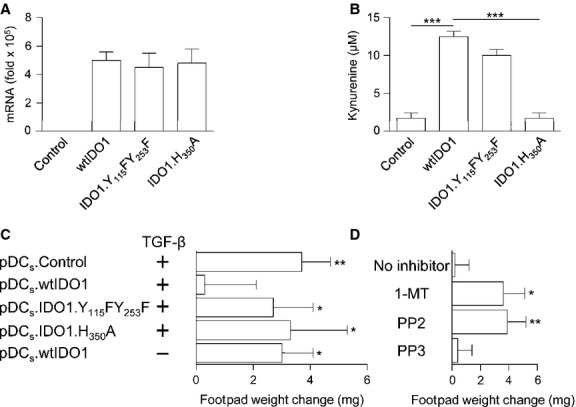
Transforming growth factor β (TGF-β) confers suppressive properties in NOD pDCs overexpressing IDO1. (**A**) NOD pDCs were transfected with mRNA coding for wtIDO1, IDO1.Y_115_FY_253_F or IDO1.H_350_A and, after 24 hrs, *Ido1* mRNA was quantified by real-time PCR by using *Gapdh* normalization. Irrelevant mRNA was used as a control (control). Data are presented as normalized transcript expression in the samples relative to normalized transcript expression in the untransfected cell culture (that is, fold change = 1, dotted line). (**B**) IDO1 catalytic activity in NOD pDCs transfected as in (**A**) was assessed as l-kynurenine in culture supernatants. (**C**) NOD pDCs were transfected as in (**A**) and treated with TGF-β prior to pulsing with IGRP and administration into recipient mice as in Figure1A. Analysis of skin reactivity of recipient mice to the eliciting peptide at 15 d is presented as change in footpad weight. (**D**) NOD pDCs transfected with wtIDO1 were treated with TGF-β in the presence or absence (no inhibitor) of 1-MT, the standard IDO1 inhibitor, PP2, a Fyn inhibitor or PP3 (negative control of PP2). After peptide pulsing with IGRP, cells were administered into recipient mice as in (**A** and **C**) and analysis of skin reactivity was performed at 15 d post-sensitization. *P < 0.05*,* **P < 0.01 and ***P < 0.001 (Student's *t*-test). Results are representative of three (**A**,**C**,**D**) and four (**B**) experiments (mean ± SD).

We evaluated whether forced expression of IDO1 in NOD pDCs would rescue the immunosuppressive ability of TGF-β. NOD pDCs were transfected with mRNAs coding for wild-type IDO1 (wtIDO1), or for IDO1 mutants lacking both ITIM tyrosine residues (IDO1.Y_115_FY_253_F) or the histidine residue required for catalytic activity (IDO1.H_350_A) 20. As a control, pDCs were transfected with an irrelevant mRNA (see Materials and methods). By means of real-time PCR, we found that, at 24 hrs of transfection, high and comparable increases in total (endogenous plus transfected) *Ido1* mRNA were observed in pDCs transfected with mRNA encoding wtIDO1, IDO1.Y_115_FY_253_F or IDO1.H_350_A, but not with the irrelevant mRNA (Fig.2A). Moreover, transfection with wtIDO1 or IDO1.Y_115_FY_253_F but not IDO1.H_350_A or irrelevant mRNA was accompanied by a significant production of l-kynurenine (Fig.2B).

On adopting an experimental model analogous to that in Figure1A*,* NOD mice were administered IGRP-pulsed cDCs in combination with pDCs pulsed with the same peptide and treated with TGF-β after transfection with mRNA encoding wtIDO1, IDO1.Y_115_FY_253_F or IDO1.H_350_A. Again, as a control, cells were transfected with irrelevant mRNA. After 2 weeks, mice were challenged with the IGRP peptide alone. Overexpression of wtIDO1 but not of IDO1 mutants or of the irrelevant mRNA resulted in TGF-β ability to activate tolerogenesis by NOD pDCs, in that the latter cells would block the *in vivo* presentation of IGRP by cotransferred immunogenic cDCs (Fig.2C). Of interest, the regulatory effects conferred by wtIDO1 on NOD pDCs were strictly contingent on exposure of TGF-β. In turn, the ability of TGF-β to confer suppressive activity on transfected NOD pDCs, made competent for both enzymic and signalling activities of IDO1, required kynurenine production and Fyn kinase activity, in that ablation of either function, namely kynurenine production and Fyn-dependent phosphorylation, would negate restoration of regulatory function in the reconstituted NOD pDCs (Fig.2D).

Therefore, our data suggest that TGF-β induction of immunoregulatory functions in pDCs requires a basal, critical expression of IDO1 protein, and that forced expression of the enzyme may compensate for the basal defect, thus making pDCs amenable to the TGF-β conditioning that involves IDO1 phosphorylation. Because the mutants lacking either phosphorylable ITIMs or the catalytic activity would not confer immunosuppressive properties on NOD TGF-β-treated pDCs, our data also suggest that a fully functional IDO1 – *i.e*., capable of both catalytic and signalling functions – is required to obviate the immunoregulatory deficit under these conditions. Why the catalytic function is a prerequisite for TGF-β induction of regulatory effects downstream of IDO1 signalling is presently unclear, but this could involve kynurenine participation in the transcriptional regulation of inflammatory cytokine gene expression by the aryl hydrocarbon receptor 32,33, for which l-kynurenine is an activating ligand 34,35.

### IDO1 overexpression combined with TGF-β treatment reprograms NOD pDCs towards a less pro-inflammatory cytokine secretion profile

**Figure 3:**
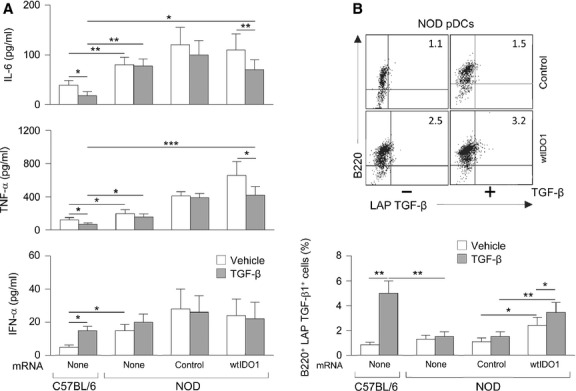
IDO1 transfection combined with transforming growth factor-β (TGF-β) treatment modulates cytokine production by NOD pDCs. (**A**) Cytokine analysis in culture supernatants. NOD pDCs were transfected with irrelevant mRNA (control) or the construct coding for wtIDO1 prior to treatment with TGF-β (at 24 hrs post-transfection). Untransfected splenic pDCs, either untreated (vehicle) or treated with TGF-β, from NOD and C57BL/6 mice were analysed for comparison. Supernatants were harvested at 24 hrs of TGF-β incubation and assayed for cytokine contents by ELISA. Results are means ± SD of four experiments. (**B**) Cytofluorometric analysis of LAP TGF-β expression. Cells from the same groups as in (**A**) were co-stained with B220– (a pDC marker) and LAP TGF-β–specific antibodies and analysed by cytofluorometric analysis. Results represent percentages of B220^+^LAP TGF-β^+^ cells. Upper panel, dot plots of most representative groups from one experiment by using NOD pDCs. Lower panel, means ± SD of three experiments. *P < 0.05*,* **P < 0.01 and ***P < 0.001 (Student's *t*-test).

To further characterize the immunoregulatory effects induced by TGF-β in NOD pDCs overexpressing IDO1, we analysed the cytokine profile of pDCs subjected to transfection with wtIDO1 or irrelevant mRNA, followed by incubation with TGF-β or medium alone for 24 hrs. Untransfected NOD and C57BL/6 pDCs, either untreated or treated with the cytokine, were used for comparison. Culture supernatants were assayed by ELISA for the presence of pro-inflammatory TNF-α and IL-6, anti-inflammatory IL-10 and IFN-α, a typical pDC cytokine that can exert either pro-inflammatory or immunoregulatory effects depending on the experimental setting 20,36. In accordance with our own 9 and others' 37 data, untransfected pDCs from pre-diabetic NOD mice released significantly higher levels of IL-6 and IFN-α as compared to C57BL/6 cells under basal, unstimulated conditions (Fig.3A). Interestingly, a similar pattern could also be observed for pro-inflammatory TNF-α. TGF-β treatment significantly reduced the constitutive, yet low production of IL-6 20 as well as that of TNF-α and increased IFN-α secretion by untransfected C57BL/6 but not NOD pDCs. However, IDO1 overexpression in combination with TGF-β treatment significantly reduced IL-6 and TNF-α secretion by NOD pDCs, albeit to a level still higher than that of C57BL/6 cell supernatants. In contrast, no modulation could be observed for IFN-α production under similar conditions (Fig.3A). Finally, no modulation was observed for IL-10 production in any groups (data not shown).

Because TGF-β is a potent immunoregulatory cytokine even when produced by DCs themselves 26,38, and because endogenous TGF-β is difficult to quantify in the presence of the recombinant protein, we measured the cytokine in the form of LAP by cytofluorometric analysis in the same cell groups as above. In accordance with our previous data 20, TGF-β treatment significantly increased the percentage of LAP TGF-β1-expressing cells in C57BL/6 pDCs (Fig.3B). In NOD pDCs, treatment with the recombinant cytokine did not increase the percentage of cells expressing the latent form of TGF-β under conditions of no transfection or transfection with control mRNA. However, wtIDO1 transfection alone of NOD pDCs led to a significant, yet limited increase in the percentage of LAP TGF-β1-expressing cells, which was further incremented when a combination of IDO1 overexpression and TGF-β treatment was used (Fig.3B).

These data thus indicate that the cytokine profile of TGF-β-treated NOD pDCs overexpressing IDO1 does not match that of cells from healthy mice, in which TGF-β is known to efficiently activate immunoregulatory IDO1 signalling 20,21. Nevertheless, in NOD pDCs, the combination of IDO1 overexpression and TGF-β treatment does induce a significant reduction in the high-level release of potent pro-inflammatory mediators, such as IL-6 and TNF-α, and it also induces up-regulation of immunosuppressive TGF-β. Similar results were obtained on studying the cytokine profile in cultures of leucocytes purified from pancreatic lymph nodes, where IL-6 and TNF-α levels were even higher than those in splenic pDCs (data not shown). Autocrine TGF-β is a marker of IDO1 signalling and is necessary to maintaining durable expression of the enzyme itself, and the long-term tolerogenic effects thereof 20,26,38. Therefore, our data suggest that TGF-β may, indeed, activate immunoregulatory IDO1 signalling in IDO1-overexpressing NOD pDCs, to an extent sufficient to turn their pronounced pro-inflammatory properties into an immunosuppressive profile.

### TGF-β up-regulates expression of endogenous *Ido1* and activates non-canonical NF-κB in NOD pDCs overexpressing IDO1

**Figure 4:**
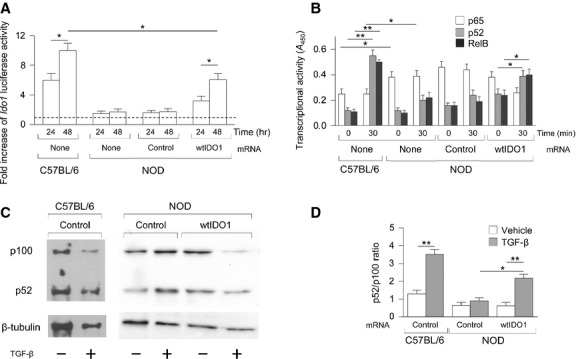
Transforming growth factor-β (TGF-β) activates the non-canonical NF-κB pathway and *Ido1* promoter in NOD pDCs overexpressing IDO1. (**A**) Time course of activation of the *Ido1* promoter in pDCs (same groups as in Fig.3) transfected with a firefly luciferase construct of the *Ido1* promoter and incubated for 24–48 hrs with TGF-β; results are normalized to the activity of a cotransfected constitutive reporter and are presented relative to those in cells not treated with TGF-β (dashed line, onefold). (**B**) ELISA of the activation of p65, p52 and RelB in nuclear extracts of pDCs not treated or treated with TGF-β for 30 min. Results are presented as absorbance at 450 nm (*A*_450_). (**C**) Immunoblot analysis of the relative expression of p100 *versus* p52. Whole cell lysates of C57BL/6 and NOD pDCs stimulated with TGF-β for 30 min. were immunoblotted with anti-p100 and anti-p100/p52 antibodies. β-tubulin was used as loading control. One representive experiment is shown. (**D**) Ratios of p52:p100 were measured by densitometric quantification of the bands detected in three experiments (means ± SD) one of which is shown in (**C**). Results are representative of three (**A**) and two (**B**) experiments (means ± SD). *P < 0.01 and **P < 0.01, cytokine-treated *versus* untreated (Student's t-test).

In IDO1 signalling, a positive feedback loop between TGF-β and IDO1 is mandatory for the maintenance of the immunoregulatory circuitry necessary for the induction of long-term tolerogenic properties in pDCs 20,24. We therefore evaluated whether the combination of IDO1 transfection and TGF-β treatment could up-regulate endogenous *Ido1* expression, similarly to endogenous TGF-β (Fig.3B). NOD pDCs were cotransfected with wtIDO1 and a plasmid construct containing the mouse *Ido1* promoter upstream of the luciferase gene, prior to incubation with TGF-β or medium alone for 24 hrs. NOD pDCs transfected with irrelevant mRNA, in addition to untransfected NOD and C57BL/6 pDCs, were used for comparison. TGF-β significantly did induce *Ido1* promoter activity in both C57BL/6 and NOD pDCs, but only when the latter cells had been transfected with wtIDO1. Nonetheless, the effect was significantly lower in NOD as compared to C57BL/6 pDCs (Fig.4A).

Our previous data demonstrated that IDO1 expression 17,25,28 and signalling 20 are contingent on the activation of the non-canonical pathway of NF-κB. The dimeric transcription factor NF-κB can be activated by the so-called canonical (classical) and non-canonical (alternative) signalling pathways, leading to distinct patterns in the composition of individual NF-κB subunits and the downstream genetic responses that are induced. The pro-inflammatory canonical pathway involves activation of the IκB kinase (IKK)β, which leads to phosphorylation-induced proteolysis of the inhibitor IκBα and consequent nuclear translocation of the p65 subunit in the form of p50-p65 dimers. In the non-canonical pathway, activation of IKKα by NF-κB-inducing kinase results in the processing of p100 to p52 and consequent formation of p52-RelB dimers, which translocate into the nucleus and activate an anti-inflammatory gene programme 17,39.

To evaluate the possible activation of non-canonical NF-κB in cells overexpressing IDO1 and treated with TGF-β, NOD pDCs, subjected to transfection with wtIDO1 or irrelevant mRNA, were stimulated with the cytokine or medium alone and, after 30 min., NF-κB family activation was measured by means of an ELISA kit specific for p65, p52 and RelB. Untransfected NOD and C57BL/6 pDCs, either untreated or treated with the cytokine, were used as controls. Significant nuclear translocation of p52 as well of RelB could be observed in both TGF-β-treated C57BL/6 and NOD pDCs, but, again, only when the latter cells had been transfected with wtIDO1. Interestingly, in basal conditions, nuclear translocation of p65 was significantly higher in untransfected NOD pDCs as compared to C57BL/6 counterparts and was not significantly modulated by TGF-β stimulation (Fig.4B). Activation of the non-canonical NF-κB pathway in NOD pDCs was further investigated by immunoblot analysis in comparison to C57BL/6 cells, for determining the relative amounts of p100 and p52. Much like in the latter cells, we found that p100 processing in p52 was significantly higher in TGF-β-treated NOD pDCs overexpressing IDO1 as compared to the other groups (Fig.4C and D). Overall, our data may provide the rationale for activating non-canonical NF-κB-dependent, immunoregulatory IDO1 signalling that leads to the expression of endogenous IDO1 in autoimmune diabetes.

## Discussion

Initially identified as a counter-regulatory mechanism in acute inflammation 40 and for its role in fetomaternal tolerance 41, IDO1 is also critical in balancing inflammation with tolerance in transplantation, cancer and autoimmunity 17,42–45. Its immunoregulatory effects are mainly mediated by DCs and depend, qualitatively, on the cytokinic milieu to which cells are exposed in the local tissue microenvironment. While acute responses are best controlled by the IFN-γ–IDO1 axis – which promotes *Ido1* transcription and thus tryptophan degradation 27 – TGF-β induces longer-term effects in pDCs, maintaining or restoring default tolerogenesis.

Transforming growth factor-β is one of the most important regulatory cytokines that contribute to establishing tolerance and preventing autoimmunity, and it acts on a broad range of hematopoietic cells – including macrophages, T cells, DCs and other immune cell types 46. In pDCs, it helps to generate and sustain the function of Tregs, through the combined effects of tryptophan starvation and kynurenines acting *via* the aryl hydrocarbon receptor of T cells 35,38 Moreover, TGF-β provides IDO1 with a non-enzymic mechanism of action, namely, signalling ability that sustains a stably regulatory phenotype in the pDCs 20 and spreads TGF-β-dependent tolerance 38.

The NOD strain of mice has become a prototypic model of human T1D 47,48. Those mice generally die of hyperglycaemia, reflecting the T cell–mediated destruction of pancreatic β cells, but they also develop a generalized autoimmune disease affecting multiple organs. There is evidence that disease susceptibility in NOD mice reflects a defect in peripheral and central tolerance 1, although the events linking defective β-cell tolerance to the T-cell compartment are poorly defined 49. Several studies have shown that NOD DCs exhibit a hyperinflammatory phenotype, and they have an elevated capacity to stimulate T cells and secrete pro-inflammatory cytokines, including IL-12 50,51. This DC phenotype can expected to directly drive differentiation of pathogenic T helper type-1 T cells and promote β-cell destruction.

Although pDCs themselves have been described as being pathogenic in T1D 16, one study has demonstrated a protective role for pDCs on transfer of naïve diabetogenic CD4^+^ T cells in NOD.Scid mice 52. pDCs could indeed prevent T1D onset, likely by inducing IDO1 production in the pancreas that inhibited the diabetogenic T cell response. In line with this, IDO1 was found to be critically involved in the induction and/or maintenance of tolerance to auto-antigens in NOD mice. Manoeuvres aimed at correcting the IDO1 defect would restore autoantigen-specific tolerogenesis 6,9. Transfection of *Ido1* into β cells prolongs graft survival in NOD mice 53. Exogenous IFN-γ is likewise protective in those mice 54. However, IDO1 activity is poorly induced by IFN-γ in DCs from pre-diabetic NOD mice 19.

In the present study, we wanted to investigate whether a global IDO1 defect occurs in NOD pDCs, and more specifically whether the defective, transcriptional response to IFN-γ – which may characterize early insulitis in pre-diabetes – is also associated with a later, IDO1-dependent defect in TGF-β–driven tolerance to auto-antigens in pDCs. A combination of peptide-pulsed CD8^−^ DCs and TGF-β–treated pDCs from pre-diabetic mice was injected into recipients to be assayed for the development of peptide-specific reactivity (Fig.1). The results showed that TGF-β failed to induce tolerizing properties in NOD pDCs. However, further experiments revealed that the defective TGF-β signalling in pDCs could be obviated by the forced expression of IDO1 in those cells (Figs4).

The ability of IDO1 overexpression to make cells responsive to tolerogenic TGF-β signalling is compatible with our previous finding that IDO1 can act as a signal transducer in pDCs through involvement of SHP-1/2 tyrosine phosphatases bound to IDO1 ITIMs domains 20. To clarify this point, we generated constructs encoding distinct IDO1 mutants. In particular, we analysed the effects of transfection either with IDO1. H_350_A, a mutant lacking the histidine residue required for catalytic activity, or with IDO1. Y_115_FY_253_F, a mutant lacking the tyrosine residues in ITIM1 and ITIM2 domains required for signalling activity. We found that loss of either catalyst or signalling function in IDO1 compromised tolerogenic TGF-β activity in pDCs made competent for WT or mutated IDO1. The requirement for catalyst activity was somewhat unexpected, and no clear explanation can be thus far provided. One possibility is that tryptophan-derived kynurenines participate in the transcriptional regulation of inflammatory gene expression, *via* engagement of the ligand-operated transcription aryl hydrocarbon receptor, which presides over the balance between pro-inflammatory and tolerogenic cytokine production in inflammatory settings 32,33. l-kynurenine, the first byproduct of tryptophan degradation, is indeed an established, endogenous ligand of that receptor 34,35. This interpretation is consistent with the finding that re-installement of pDC immunosuppressive potential requires IDO1's competence for both enzymic and signalling activities, suggesting that some cooperative factor seems to be missing in NOD mice reconstituted selectively for IDO1's signalling. This could be because of the lack of tryptophan catabolites, which originating from IDO1's enzymic activity, act as AhR ligands, necessary for triggering strong Treg-dependent responses 34,35.

The analysis of cytokines produced by pDCs from NOD mice, and by pancreatic leucocytes as well, reflected the immunogenic phenotype observed *in vivo*, those cells featuring a dramatic expression of IL-6 and TNF-α and low autocrine TGF-β, as compared to WT C57BL/6 counterparts. Although TGF-β alone could not induce significant immunomodulatory effects in NOD pDCs, the forced overexpression of IDO1 allowed the cytokine to correct, at least partially, the hyperproduction of pro-inflammatory cytokines and restored autocrine production of TGF-β necessary to maintaining immunoregulatory IDO1 signalling and long-term IDO1 expression.

In summary, our data demonstrate that not only the enzymic function, but also its signalling ability are defective in NOD mice, therefore contributing to autoimmunity in at least two different ways. The TGF-β–driven default tolerogenesis which fosters tolerance to self 26,38 may be basically compromised in the absence of functional IDO1, thus predisposing NOD mice to autoimmunity in general 1. Along this line, data not included in the present manuscript have shown that an additional defect to be observed in NOD pDCs lies in poor basic expression of SHP-1 and SHP-2 tyrosine phosphatases, which defect is not alleviated by TGF-β. Lack of reinforcement of IDO1 expression by IFN-γ, which would otherwise accompany and mitigate local inflammation as a negative feedback mechanism, may represent a superimposed condition that allows for persistence and progression of the inflammatory state, as may be the case for the transition from early insulitis to β-cell destruction. Both defects could be traced to aberrant *Ido1* transcription, and this, in turn, could be a consequence of multiple defects in signalling events upstream of *Ido1* transcription 6,19. Forced IDO1 expression rescues both activities – enzymic and signalling – of the protein, thus providing proof-of-principle that a global IDO1 defect predisposes NOD mice to autoimmunity.
